# RETRACTION: Chitoheptaose Promotes Heart Rehabilitation in a Rat Myocarditis Model by Improving Antioxidant, Anti‐Inflammatory, and Antiapoptotic Properties

**DOI:** 10.1155/omcl/9864523

**Published:** 2026-03-31

**Authors:** Oxidative Medicine and Cellular Longevity

RETRACTION: Q. Zhao, L. Yin, L. Zhang, D. Jiang, L. Liu, H. Ji, “Chitoheptaose Promotes Heart Rehabilitation in a Rat Myocarditis Model by Improving Antioxidant, Anti‐Inflammatory, and Antiapoptotic Properties,” *Oxidative Medicine and Cellular Longevity*, 2020, 2394704, https://doi.org/10.1155/2020/2394704.

The above article, published online on 11 April 2020 in Wiley Online Library (wileyonlinelibrary.com), has been retracted by agreement between the authors; the journal Editor‐in‐Chief, Jeannette Vasquez‐Vivar, and John Wiley & Sons Ltd. The retraction has been agreed due to errors in the study design (concentrations of DP of COS) being identified by the authors, leading to incorrect conclusions being stated in the article.

After assessment by the Chief Editor, the results and conclusions of the article are no longer reliable, and it is therefore retracted.

